# Diets and Feeding Practices during the First 1000 Days Window in the Phnom Penh and North Eastern Districts of Cambodia

**DOI:** 10.3390/nu10040500

**Published:** 2018-04-18

**Authors:** Somphos Vicheth Som, Sophonneary Prak, Arnaud Laillou, Ludovic Gauthier, Jacques Berger, Etienne Poirot, Frank T. Wieringa

**Affiliations:** 1 Department of Fisheries Post-Harvest Technologies and Quality Control, Fisheries Administration, 186 Preah Norodom Boulevard, Phnom Penh 12101, Cambodia; somphosvichethsom@gmail.com (S.V.S.) gauthier.ludo@hotmail.fr (L.G.); 2 National Nutrition Program, Ministry of Health, 31A Rue de France (St. 47), Phnom Penh 12202, Cambodia; sophonprak@gmail.com; 3 United Nations Children’s Fund Cambodia, Department of Child Survival and Development, 19&20 street 106, Exchange Square Building, Phnom Penh 12101, Cambodia; epoirot@unicef.org; 4 Institut de recherche pour le dévelopment, IRD/Université de Montpellier/SupAgro, 911, avenue d’Agropolis, 34394 CEDEX 5 Montpellier, France; jacques.berger@ird.fr (J.B.); franck.wieringa@ird.fr (F.T.W.)

**Keywords:** women nutrition, exclusive breastfeeding, complementary diet, policy, strategy, early childhood development, Cambodia

## Abstract

Although several health and development indicators have improved significantly in Cambodia, inadequate breastfeeding and inappropriate complementary feeding practices leave many children at high risk of malnutrition during the early stages of life. In 2014, the prevalence of wasting and stunting among Cambodian children under 5 were 10% and 32%, respectively. Thus, a strong focus on improving feeding practices within the first 1000 days window to reduce child malnutrition prevalence in Cambodia is needed. This cross-sectional study assessed the current feeding practices among of women of reproductive age, pregnant women, lactating women and children less than 24 months living in six districts from Phnom Penh and two rural provinces in the North East of Cambodia. The nutritional status of pregnant women was poor, with 21.4% having a Middle Upper arm circumference below 23 cm. While breastfeeding was predominant within the first 6 months of age in every district, feeding practices of pregnant women and children were a concern, as >70% of the children were not meeting the minimum acceptable diet, and most of the women did not improve their diet during pregnancy. Inadequate nutrition during the first 1000 days is highly prevalent in Cambodia. A comprehensive national Mother, Infant and Young Child Nutrition strategy needs to be developed and operationalized to improve feeding practices of Cambodian women and children.

## 1. Background

Cambodia has been on a positive trajectory for a number of health and development indicators [1]. To be able to achieve the country’s 2030 Sustainable Development Goals (SDGs) and improve the health of the Cambodian children and women, there is an urgent need to accelerate efforts related to nutrition. Poor breastfeeding and complementary feeding practices put infants and young children at high risk of malnutrition very early in life. Paramount to the success of those efforts are the critical feeding practices during the 1000 days period. Cambodia’s Infant and Young Child Feeding (IYCF) program is one of the most important components of the National Nutrition Program (NNP)’s Fast Track Road Map for Improving Nutrition [2].

NNP and its partners have worked closely to achieve the IYCF goals set by UNICEF (United Nations Children’s Fund) and the WHO (World Health Organization). Since 2000, the national IYCF program has improved notably. For example, training and educational materials in different forms and channels related to IYCF have been developed and used nation-wide by different agencies, both governmental and non-governmental. The promotion of breastfeeding has been one of the most significant public health success stories for Cambodia, with rates of exclusive breastfeeding and early initiation of breastfeeding increasing significantly among all groups in the period 2000–2010 [3]. Unfortunately, during the same period, the use of breast-milk substitutes and bottle feeding has also increased among children above 6 months of age [3].

The 2014 Cambodian Demographic Health Survey (CDHS) demonstrated a new decline in the prevalence of exclusive breastfeeding under 6 months of age (from 75% to 65% from 2010 to 2014 [1]). In addition, from 2010 to 2014 [4], the prevalence of new-borns receiving pre-lacteal feeding had increased by 8.6% from 19.1% to 27.7%. Worryingly, it had almost doubled in urban areas, with over 50% of urban new-borns receiving pre-lacteal feeding in 2014. Giving an infant water or milk-based pre-lacteal food delays the child’s first consumption of breast milk, depriving the infant of the many benefits of the colostrum and breastfeeding. Research has shown that children who received pre-lacteal feeding were 3.9 times more likely to be consuming a breast-milk substitute than those who did not [5], and therefore, to not be following the international guidelines of exclusively breastfeeding until the age of 6 months.

Despite the country’s economic growth, the quality of young children’s (6–23.9 months) diet remains a concern, too. More than 60% of children aged 12 to 23.9 months and up to 80% of children aged 6 to 8 months do not receive the minimum acceptable diet daily [4]. The poorest children and children living in rural areas were, respectively, 4 and 2 times less likely to receive the minimum acceptable diets than were children from the wealthiest families or urban children [4]. This incapacity to provide adequate nutrition to young children (6–23.9 months) has a direct impact on their micronutrient status and their growth. In addition, the nutritional status of a woman before and during pregnancy is also important for a healthy pregnancy outcome [6] and the early development of the future child. While a lot of women and men know what women should do during pregnancy, including eating healthy foods and four antenatal care visits, they do not know what constitutes healthy foods for pregnant women, how much to consume, and the importance of weight gain during pregnancy [7]. This may be due to women not getting proper nutrition counseling during antenatal care visits (ANC) at health centers.

Therefore, interventions focusing on prevention of malnutrition [8], such as ensuring that pregnant and lactating mothers are adequately nourished, and children receive the appropriate feeding, could help decrease the high prevalence of stunting and wasting observed in sub-regions of Cambodia such as in the North East [9].

The present study assessed the feeding practices of women of reproductive age, pregnant women and children less than 24 months of age in Phnom Penh and in 2 provinces in the North East. The secondary objective of the study was to inform the government of Cambodia on those practices in order to develop a comprehensive national infant and young child feeding strategy to ensure appropriate growth.

## 2. Material and Methods

### 2.1. Data Sources

Interviews with mothers were conducted in Phnom Penh (Russei Kaev district), Kratie province (Chitr Borie and Krong Kratie districts), and Ratanakiri province (Ou Chum, Krong Ban Lung and Bar Kaev districts) at baseline as part of a project called “MyHealth”. The main objective of this project is to collect in-depth data over 3 years on the health and nutritional status of the selected UNICEF districts in the 3 provinces to better inform the government on progress that can be made with enhanced health monitoring. A sample size of 1200 children under 2 years of age per site was calculated, exhibiting a reduction in child stunting from 32% to 26% over a 3–5-year period (with a precision of 3% and a dropout of 20%), with all pregnant women from the selected villages being included. A list of children under 2 years and pregnant women was obtained from midwives and village health volunteers covering all the villages. Subsequently, households with children under 2 years old were randomly designated to the study, together with siblings.

Data on dietary practices during the 1000 days windows were collected by experienced and trained teams that visited a total of 927 households with a pregnant woman and 4161 households with children under 2. The teams collected information on: (i) socio-economic data; (ii) health knowledge; (iii) diet, including dietary diversity of children and women, breastfeeding diet; and (iv) mother and child anthropometry. Data for children aged between 0 and 24 months (*n* = 4161) and women (pregnant and non-pregnant) (*n* = 4072) were used for the present study.

### 2.2. Outcomes Measured

The household wealth index is a composite measure of a household’s living standard that was calculated through principal component analysis, as described by Filmer and Pritchett [10]. It gathers information regarding the accessibility and type of water and sanitation facilities, materials used for housing construction, type of fuel used for cooking, and ownership of selected assets such as a radio, television, refrigerator. The wealth index was then divided into quintiles.

Anthropometric measurements were collected in triplicate from each child under 2 years to ensure accuracy. Children’s recumbent lengths or standing heights were measured to the nearest 1 mm. The nutritional status of children was defined by means of the height-for-age, weight-for-height, and weight-for-age z-scores, calculated according to the Child Growth Standard of the World Health Organization (WHO) using the WHO Anthro software (WHO, Geneva, Switzerland). Z-scores below −2 for length/height-for-age (HAZ), weight-for-length/height (WHZ), and weight-for-age (WAZ) were defined as stunting, wasting, and underweight, respectively. To ensure the accuracy of the data, extreme values were excluded from the analysis: weight-for-age z-score < −6 or > 5; length/height-for-age z-score < −6 or > 6; weight-for-length/height z-score < −6 or > 6. Excluded values represented less than 5% of the total values. Body mass index (BMI) of women of reproductive age [11] and middle upper-arm circumference of pregnant women [12] were measured.

Infant and Young Child Feeding was assessed following UNICEF and WHO indicators on appropriate breastfeeding and IYCF practices [13]. Complementary feeding indicators were based on the following seven food groups: (1) grains, roots, tubers; (2) legumes and nuts; (3) vitamin A fruits and vegetables; (4) other fruits and vegetables; (5) meats; (6) eggs; and (7) dairy products [13]. The minimum meal frequency indicator was created from including breastfed children who had received solid, semi-solid or soft foods the minimum number of times or more and non-breastfed children who received solid, semisolid or soft foods or milk feeds the minimum number of times or more the previous day [13]. The WHO guidelines were also used to construct an overall dietary adequacy indicator. Thus, for breastfed infants, the minimum acceptable diet included those children who had at least the minimum dietary diversity and the minimum meal frequency. For non-breastfed children to reach minimum dietary adequacy, they should, in addition to these criteria, have consumed at least 2 milk products over the day, such as infant formula, cow milk or other animal milk [13]. Dietary diversity in women was measured through the Women’s Dietary Diversity Score (WDDS). WDDS is based on nine food groups, as described by FAO (2011) and selected for use e.g., the U.S. Agency for International Development (USAID) Feed the Future and the World Food Programme (WFP) Food for Peace development food assistance programmes [14]. It is a simple sum of scores of the 9 categorized food groups, ranging from 0 to 9. Lower values for WDDS indicate nutritionally inadequate dietary diversity. The nine food group indicators are starchy staples, legumes and nuts, dairy, organ meat, eggs, flesh meat and fish, dark green leafy vegetables, other vitamin A-rich vegetables and fruits, and other fruits and vegetables.

### 2.3. Statistical Analysis

The prevalence in dietary adequacy indicators and several children characteristics were analyzed using chi-square test. Nutritional status indicators and the Woman Dietary Diversity Scores (WDDS) between the regions were compared using Kruskall-Wallis test, as well as pregnant women Middle Upper arm circumference (MUAC) distributions among Dietary Diversity Score groups (1–2, 3–4, 5+) within each region.

To study the evolution of the child’s feeding practices across age, we created a categorical variable with 5 categories: (i) children who are exclusively breastfed; (ii) breastfed children who drink additional liquids other than milk products; (iii) breastfed children who drink any additional liquids (water, milk products, juice, etc.); (iv) breastfed children who consume any additional liquids and semi solid foods; and (v) non-breastfed children. To see how the conditional distribution of this variable changes over age, we computed conditional densities and the associated conditional density plots. The same method was used to test how exclusive breastfeeding and introduction of soft, solid and semi-solid food changes over child’s age.

All the analyses were performed with STATA software version 13.1 (StataCorp LLC., College Station, TX, USA) and R software version 3.4.0 (https://www.r-project.org/).

Ethical approval for the study was obtained from the Cambodia National Ethical Committee for Health Research. Informed consent was obtained from all participants, with consent obtained from parents or guardians for participating children.

## 3. Results

In total, 4161 children aged between 0 and 24 months and 4072 women (pregnant and non-pregnant) were recruited to the study (Table 1). Nutritional status, socio-economic status and education were significantly different between the provinces, with children and women living in Phnom Penh scoring higher than those living in Kratie or Ratanakiri. Among women, 72% were lactating, while 23% were pregnant. Nutritional status of the pregnant women was poor, with 21.4% having a MUAC below 23 cm, indicating malnutrition. The Body Mass Index showed up the issue of the double burden in urban areas, as 14.4% of the women were underweight, while 23.4% were overweight.

**Figure 1:**
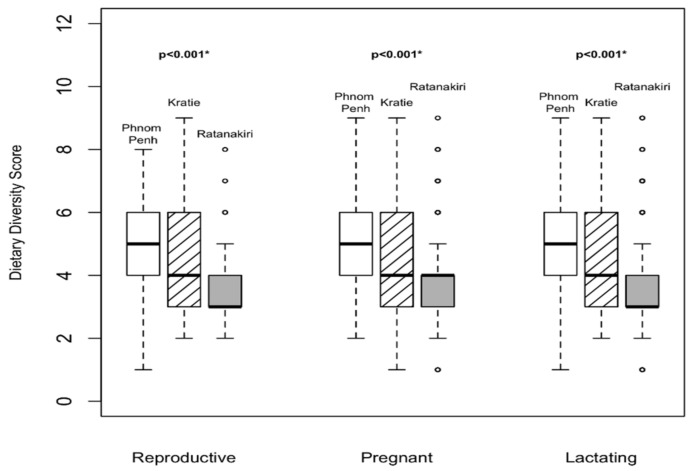
Comparison of women’s dietary diversity scores between regions and physiological status. *: Kruskal-Wallis test performed; ◦: out layers.

**Figure 2:**
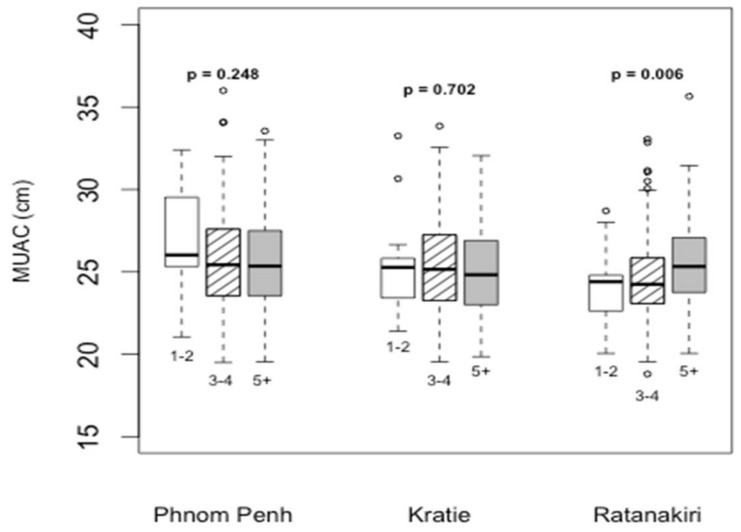
Comparison of pregnant women’s MUAC (Middle Upper arm circumference) according to their dietary diversity score and regions. (Note: *p* < 0.05 shows a significant difference in the mean of MUAC within provinces between the 3 categories of WDDS (Women’s Dietary Diversity Score): (i) 1–2 food groups consumed; (ii) 3–4 food groups; (iii) above 4 food groups; ◦: out layers).

For the feeding practices of women, the relevant women’s dietary diversity score (WDDS) and interquartile ranges are presented in Figure 1. Overall, WDDS was better in Phnom Penh than in Ratanakiri. In Ratanakiri, MUAC was positively associated with a higher diet diversity score in pregnant women (*p* = 0.006), while no difference was observed in either Kratie or Phnom Penh (Figure 2).

**Figure 3:**
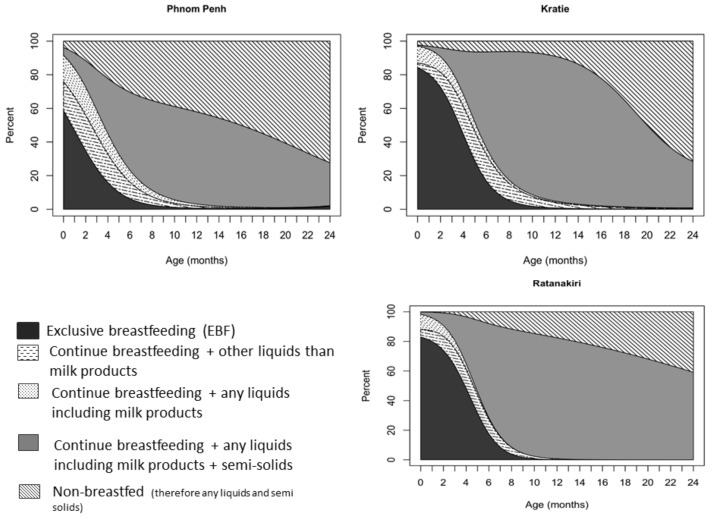
Infant feeding practices by age (note: BF: continued breastfeeding).

**Figure 4:**
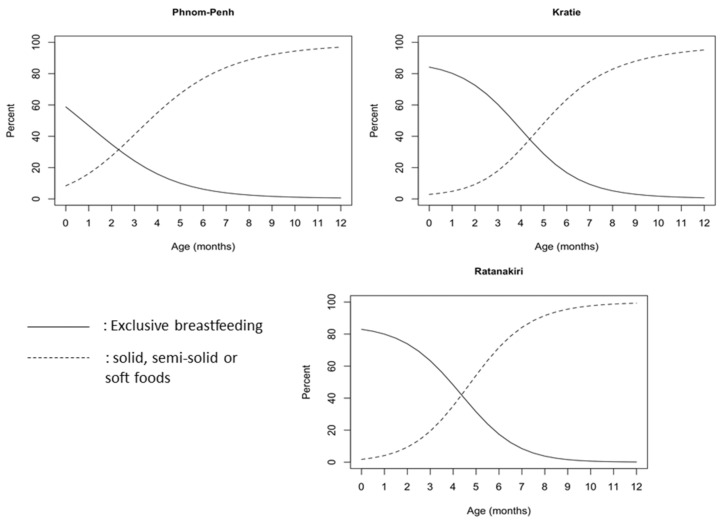
Percentage of infants exclusively breastfed (EBF) and percentage receiving liquids, solid, semi-solid or soft foods.

At the age of 1 month, 46.8% of the children in Phnom Penh, 80% in Kratie and Ratanakiri were being exclusively breastfed. This prevalence continued to decrease, as at 3 months, only 24.2% in Phnom Penh, 60.3% in Kratie and 63.3% in Ratanakiri were still following the international guidelines for breastfeeding. In Phnom Penh, only 7.9% of the infants were still exclusively breastfed at 5.5 months of age, while in Kratie and Ratanakiri, the prevalence was 22.1% and 23.9%, respectively (*p* < 0.001 for difference with Phnom Penh). Among 6-month-old children, as many as 30% of the children in Phnom Penh were no longer breastfed, while only 6% in Kratie and Ratanakiri were not breastfed (Figure 3). Around 35% of infants under the age of 3 months received water or other liquids with milk in Phnom Penh, while this prevalence was 22% in Kratie and 17% in Ratanakiri. From the age of 20 months onwards, we observed a significant reduction in children still being breastfed. Approximately 50% were not breastfed anymore in Kratie (Figure 3), around 60% in Phnom Penh, and 35% in Ratanakiri. In Phnom Penh, only 27.6% of the children continued to be breastfeed until 2 years of age, a percentage that was comparable with that in Kratie (28.5%), but much lower than that in Ratanakiri (59.1%). Timely introduction of complementary feeding (from the age of 6 months, Figure 4) was recorded for 77–89%, 64–83% and 72–92% of children aged 6–8 months in Phnom Penh, Kratie and Ratanakiri, respectively, with children being given solid, semi-solid, or soft food.

Overall, <25% of Cambodian children aged 6–24 months in the study districts met the minimum acceptable diet (Table 2). In the selected districts of Phnom Penh and Kratie, breastfed children had a significant better prevalence of meeting the minimum acceptable diet, while this was the opposite in Ratanakiri. The most common problem with feeding practices was an inadequate number of food groups (Minimum Diet Diversity); only 33.8% of children received foods from the minimum number of food groups for their age in Phnom Penh, in comparison to 22.1% in Kratie and 29.4% in Ratanakiri. In every province, breastfed children always had a higher prevalence of children receiving the minimum meal frequency and the minimum acceptable diet than non-breastfed (*p* < 0.01). No difference was observed between gender, except in Ratanakiri, where 76.6% of the girls had the minimum meal frequency, compared to 83.4% of the boys (*p* < 0.019). Wealth was also a strong contributor to the difference of feeding practices in Ratanakiri, as the prevalences of children receiving the minimum dietary diversity and the minimum acceptable diet were always the lowest among the poorest population (*p* < 0.001).

## 4. Discussion

To our knowledge, this is the first study reporting a significant number of pregnant Cambodian women, around 20% (18% in Phnom Penh, 22.5% in Kratie and 22.9% in Ratanakiri), with a low MUAC, indicating malnutrition. Cambodian women in the studied districts had poor eating habits and did not improve the diversity of their diet when becoming pregnant. Malnutrition in pregnancy is known to increase the risk of low birth weight [12] and early stunting, which might affect optimal development during childhood [15]. Poor eating habits have several causes in Cambodia, including, for example, women tending to compromise on food purchases to save money for childbirth expenses [16]. Additionally, ‘eating well’ in pregnancy typically means increasing intake of routinely consumed foods, namely rice, as opposed to seeking foods with a higher protein and micronutrient content, such as meat and eggs [16]. Moreover, according to recent NNP/UNICEF/Helen Keller International (HKI) [17] formative research, pregnant women, especially in Phnom Penh, limit the amount and type of food they eat to ensure that the baby does not grow ‘too big’ to guarantee an ‘easy’ delivery and postpartum recovery. The formative research implemented in 2016 confirmed the finding of the present study that pregnant women have limited dietary diversity. Other dietary behavior reported from rural areas was also of concern for the expected delivery. In rural areas, pregnant women followed traditional beliefs to ensure an easy, fast and inexpensive (natural) delivery characterized by “*Eat fast and finish before your husband so your delivery will also be fast*”. Pregnant and lactating women are particular vulnerable to malnutrition because of higher nutrient demands during pregnancy and lactation [18]. Gender disparities also potentially influence maternal undernutrition. Culturally, especially in poor families, men get the best food at mealtimes and eat before children and women, who serve themselves last. This can lead not only to both maternal undernutrition, but also impacts adolescent girls and their reproductive health later in life [19].

The targeted districts showed a high prevalence of predominant breastfeeding within the first 6 months of age ranging from >90% in the North East to close to 70% in Phnom Penh. Unfortunately, the prevalence of children receiving exclusively breast milk at the age of 5.5 months was very low (<20%), as most of them were receiving other fluids including water. A recent survey reported that 74% of the liquids fed in a bottle were water [20]. It is therefore essential to promote better practices for exclusive breastfeeding in Cambodia. Also, common beliefs are reducing the number of infants who receive appropriate breastfeeding within the first hour of life. Mothers believe that they do not have any, or not enough, breast milk, and therefore include a can of formula in their bag for the hospital. Despite the high level of awareness about the benefits of breast milk in urban areas, anxiety over not being able to satiate the ‘hunger’ cry of the baby with only breast milk and loosing face in front of senior people such as doctors triggers mothers to provide formula milk at an early age [17]. Once delivered, practices at the hospital level continue to jeopardize early breastfeeding. From 2010 to 2014, the prevalence of new-borns receiving pre-lacteal feeding increased by 8.6 per cent [3]. It almost doubled in urban areas, reaching more than 50 per cent in 2014 [1]. It is thus important to refocus and reinforce the message to health practitioners to promote correct exclusive breastfeeding practices.

The weak enforcement of Sub-Decree 133 on Marketing of Products for Infant and Young Child Feeding, and the growing promotion and sales of breast-milk substitute products, are also contributing factors to the decline in breastfeeding rates during the first 6 months [3]. Worryingly, several studies on the microbiological quality of drinking water have reported high levels of contamination in our selected districts (Personal communication). Ingestion of water with bacterial contamination, including fecal pathogens, can cause diarrhea [21] and increases the risk of stunting and malnutrition. The addition of water at an early stage is therefore an important preventable risk for stunting and wasting in Phnom Penh and the North-East provinces of Cambodia.

Keeping exclusive breastfeeding as the social norm is also being hampered with Caesarean-sections becoming a trend, especially amongst the upper and middle class in urban and peri-urban areas [17]. Many Cambodian women believe there is a clear connection between what they eat and drink (including medication) and the quality of their breast milk. To prevent the new-born from exposure to the medication used during Caesarean-sections, mothers avoid breastfeeding directly after delivery and the first days post-partum, thereby denying the new-borns access to colostrum. This calls for every health facility providing maternity services and care for new-born infants to provide support to mothers in breastfeeding using the ten steps of the baby-friendly hospital initiative (BFHI) and to go beyond by ensuring that breastfeeding indicators are part of a system of hospital assessment and accreditation.

Complementary feeding was introduced from 6 months onwards for around 75% of our study population. However, >80% of the children did not receive the minimum acceptable diet, showing that the quality of infants’ and young children’s (6–24 months) diet is still a concern in Cambodia. Indeed, dietary diversity was the main reason, rather than the number of meals, for not meeting international guidelines for the minimum acceptable diet. This limited capacity to provide the optimal diet to the children is jeopardizing the intellectual, mental and physical growth of the children, which can lead to poor cognitive development and limited opportunities for work throughout life. Our results are in line with the 2014 CDHS [1], which also reported a low percentage of children receiving the minimal acceptable diet. Lack of knowledge regarding nutritious foods is one concern. A Cambodian formative research observed that the majority of Cambodians think that rice is nutritious enough to avoid a baby from becoming ill and support weight gain. Additionally, mothers who work tend to compensate for the lack of solid foods with extra formula milk [17]. Even though the Communication for Behavioral Impact (COMBI) strategy developed between 2000 and 2014 has been very successful in terms of awareness [17] due to successful interventions such as popular TV commercials and NGO community programs, only a few mothers are currently implementing the appropriate feeding practices in our selected districts.

Developing better awareness of the importance of food diversity and nutritious foods, and putting efforts into communicating the appropriate diversity of food that children need at each step of their development is key.

## 5. Conclusions

In view of the findings of the present study, it is essential to reinforce several messages, starting at least from pregnancy, but preferably before conception, related to the feeding practices for children of up to 2 years of age. It is time to leverage the perception that breast milk is more convenient, more hygienic, cheaper and aspirational than formula milk. A new trend should be created that positions breastfeeding practices and appropriate complementary feeding as a new status symbol and as the right behavior for good parenting practices. The strategy should also be extended to pregnant women who have not been a targeted group in previous campaigns. The continuum of care and feeding and feeding practices is essential, in order not to limit the potential impact of the next national campaign.

For all of these reasons, there is an urgent need to develop a comprehensive national mother, infant and young child nutrition strategy comprising a communication strategy and associated action plan that focuses on improving IYCF practices in both rural and urban settings through consistent and context-adapted messaging, effective use of resources, and improved coordination between agencies and government ministries. Changes must become an aspiration and not a directive. The strategy will set out priority areas for communication support and interventions to harmonize approaches across government ministries and development partners and create an enabling environment for improved mother, infant and young child nutrition behaviors among the Cambodian population.

Finally, initiatives to develop a system of hospital assessment and accreditation are needed beyond BFHI, and this would require close collaboration between different actors (health and nutrition experts) to ensure that breastfeeding and other indicators are part of the certification arrangement.

## Figures and Tables

**Table 1:** Characteristic of the sample.

Characteristics	Phnom Penh (%)	Kratie (%)	Ratanakiri (%)	Chi-2 ****
Children				
Height for age z-score	*n* = 1346	*n* = 1453	*n* = 1362	
HAZ < −3	2.6	4.3	7.0	*p* < 0.001
−3 ≤ HAZ < −2	8.6	11.9	20.8	
−2 ≤ HAZ	88.8	83.8	72.1	
Weight for height z-score	*n* = 1277	*n* = 1453	*n* = 1362	
WHZ < −3	1.9	4.3	0.9	*p* < 0.001
−3 ≤ WHZ < −2	8.5	15.0	11.7	
−2 ≤ WHZ	89.6	80.7	87.5	
Wealth index ***	*n* = 1130	*n* = 1021	*n* = 1253	
Poorest	6.5	31.0	23.7	
Poorer	10.3	27.2	28.1	*p* < 0.001
Middle	22.6	22.9	24.2	
Richer	22.8	11.9	11.0	
Richest	37.9	7.0	13.0	
Mother Education	*n* = 1018	*n* = 1015	*n* = 1253	
no Education/Informal	12.9	17.6	44.1	*p* < 0.001
Primary	37.3	48.9	33.4	
Secondary and more	49.8	33.5	22.5	
Women				
BMI (kg/m^2^) *	*n* = 863	*n* = 1189	*n* = 1093	
Underweight	14.37	16.48	17.93	*p* < 0.001
normal	62.22	69.47	72.28	
Overweight	19.24	12.36	9.06	
Obese	4.17	1.68	0.73	
MUAC (cm) **	*n* = 271	*n* = 381	*n* = 275	
<21 cm	3.69	2.1	4	*p* < 0.001
21 ≤ MUAC < 23	14.39	20.47	18.91	
>23	81.92	77.43	77.09	
Wealth index ***	*n* = 1134	*n* = 1570	*n* = 1368	
1st Quintile	5.91	27.64	24.42	
2nd Quintile	8.02	24.27	26.1	*p* < 0.001
3rd Quintile	22.22	16.82	19.3	
4th Quintile	29.1	19.24	16.59	
5th Quintile	34.74	12.04	13.6	
Mother Education	*n* = 784	*n* = 1178	*n* = 1016	
no Education/Informal	12.37	17.49	46.75	
Primary	40.31	48.47	33.86	*p* < 0.001
Secondary and more	47.32	34.04	19.39	

Note: * measured for all women except pregnant; ** measured for pregnant women; *** wealth index gathers information regarding the accessibility and type of water and sanitation facilities, materials used for housing construction, type of fuel used for cooking, and ownership of selected assets such as a radio, television, refrigerator. The wealth index was then divided into quintiles; **** *p* < 0.05 shows a significant difference within sub-groups between provinces. HAZ: Z-scores below −2 for length/height-for-age; WHZ: Z-scores below −2 for weight-for-length/height; BMI: body mass index; MUAC: Middle Upper arm circumference.

**Table 2:** Infant and young child feeding practices.

	Phnom Penh Districts						Kratie Districts						Ratanakiri Districts					
Background Characteristic	Minimum Dietary Diversity		Minimum Meal Frequency		Minimum Acceptable Diet		Minimum Dietary Diversity		Minimum Meal Frequency		Minimum Acceptable Diet		Minimum Dietary Diversity		Minimum Meal Frequency		Minimum Acceptable Diet	
	*n*	%	*n*	%	*n*	%	*n*	%	*n*	%	*n*	%	*n*	%	*n*	%	*n*	%
Age																		
6–11	339	28.6	328	59.1	328	14.9	414	17.1	365	75.9	365	14.5	329	21.9	289	81.3	289	20.4
12–17	305	36.7	303	53.8	302	22.2	316	23.4	299	69.6	299	18.1	345	33.9	324	79.3	324	27.8
18–24	213	38.0	209	46.9	209	19.6	274	28.1	259	50.2	259	11.2	225	33.3	211	78.2	211	23.2
***p***	***0.031***		***0.021***		***0.059***		***0.003***		***0.0001***		***0.072***		***0.001***		***0.674***		***0.098***	
Breastfeeding																		
Non-breastfed	394	34.3	394	18.5	393	6.1	230	33.5	219	29.7	219	8.7	191	47.6	182	69.8	182	33.5
Breastfed	451	33.0	446	85.7	446	29.8	774	18.7	704	78.1	704	16.6	708	24.4	642	82.6	642	21.3
***p***	***0.707***		***0.0001***		***0.0001***		***0.0001***		***0.0001***		***0.002***		***0.0001***		***0.0001***		***0.001***	
Wealth Index																		
Poorest	123	27.6	120	51.7	110	15.8	430	17	390	71.5	390	13.8	413	22.3	374	78.1	374	17.6
Middle	172	37.2	167	68.3	167	24.6	157	21	143	82.5	143	18.2	196	33.2	176	76.7	176	27.3
Richest	458	34.5	450	49.1	450	18.0	129	23.3	121	83.5	121	20.7	223	39.5	211	86.3	211	33.6
***p***	***0.21***		***0.0001***		***0.12***		***0.215***		***0.003***		***0.15***		***0.0001***		***0.027***		***0.0001***	
Mother Education																		
No education	90	25.6	88	47.7	88	14.8	129	9.3	123	66.7	123	7.3	340	19.7	304	78.6	304	15.5
Primary	249	31.7	167	60.6	246	19.1	346	22.3	314	77.7	314	17.8	290	33.8	264	78.4	264	28.4
Secondary	339	38.9	450	53.9	330	23.0	236	19.5	213	79.8	213	18.8	202	39.6	193	84.5	193	32.6
***p***	***0.03***		***0.08***		***0.176***		***0.006***		***0.021***		***0.12***		***0.0001***		***0.204***		***0.0001***	
Gender																		
Male	382	32.7	375	57.3	375	20.5	370	18.1	336	78.9	336	16.1	417	30.5	385	83.4	385	26.8
Female	371	35.3	362	50.3	362	17.7	346	19.9	318	73.3	318	16	415	28.4	376	76.6	376	21.8
***p***	***0.454***		***0.055***		***0.324***		***0.532***		***0.093***		***0.991***		***0.522***		***0.019***		***0.112***	
TOTAL	*857*	*33.8*	*840*	*54.2*	*839*	*18.7*	*1004*	*22.1*	*923*	*66.6*	*923*	*14.7*	*899*	*29.4*	*824*	*79.7*	*824*	*24*

Note: *p* < 0.05 shows a significant difference in the prevalence of Minimum Dietary Diversity (MDD), Minimum Meal Frequency (MMF) or Minimum Acceptable Diet (MAD) within province between sub-characteristic (age, breastfeeding, wealth index, mother education, gender).
